# Reversible Second-Degree Heart Block Attributed to Ondansetron: A Rare Side Effect

**DOI:** 10.7759/cureus.64109

**Published:** 2024-07-08

**Authors:** Abey Sebastian, Emmanuel Bhaskar, Swathy Moorthy, Lakshmi M

**Affiliations:** 1 Internal Medicine, Sri Ramachandra Institute of Higher Education and Research, Chennai, IND; 2 General Medicine, Sri Ramachandra Institute of Higher Education and Research, Chennai, IND

**Keywords:** bradycardia, mobitz heart block, adverse effect, ondansteron, primigravida

## Abstract

A primigravida at 36 weeks with gestational diabetes mellitus and hypothyroidism and no prior chronic medical illness was admitted for safe confinement. A cesarean section was required to deliver the baby with breech presentation complicated by a slow progression of labor. Asymptomatic sinus bradycardia with a heart rate of 40 per minute was observed during the induction of anesthesia. Before bupivacaine administration for spinal anesthesia, she was administered pantoprazole 40 mg and ondansetron 4 mg intravenously. ECG recording showed a type 1 Mobitz second-degree heart block. Follow-up ECG showed progression of heart block to type 2 Mobitz second-degree heart block. The second-degree heart block persisted for 16 hours, during which the patient was asymptomatic, and the ventricular rate was maintained at a range of 60-80 per minute. After normalization of rhythm, the patient was observed in the ICU. She received another dose of ondansetron 4 mg intravenously for vomiting, and the heart block recurred. The rhythm disturbance was attributed to ondansetron. Her rhythm normalized after 36 hours, and she was subsequently discharged home three days later.

## Introduction

Nausea and vomiting are prevalent medical manifestations of pregnancy, impacting as many as 80% of all pregnancies to some extent. While symptoms typically subside by the 16th week of pregnancy, 20% of women may experience persistent symptoms during the pregnancy. Although it impacts less than 1% of women, severe hyperemesis gravidarum can be extremely incapacitating, necessitating hospitalization and rehydration of fluids in its most severe form [1]. Ondansetron, a selective antagonist of the 5HT3 serotonin receptor, is commonly prescribed to prevent postoperative nausea and vomiting. It has been demonstrated that 5HT3 receptor antagonists induce ECG alterations in healthy individuals [2]. These varied from an extended QTc interval and reduced heart rate observed with ondansetron to an extended PR interval, prolonged QRS complex duration, and increased heart rate observed with dolasetron [3]. It is frequently associated with the development of rhythm disturbances. Some of the dysrhythmias associated with its use include bradycardia, ventricular tachycardia, atrial fibrillation, ST-T changes, and ventricular bigeminy [4]. The FDA of the United States issued a series of safety communications concerning ondansetron and the potential for protracted QT intervals to result in torsades de pointes between 2011 and 2012. In the end, the FDA mandated modifications to the prescribing information for ondansetron, which included the elimination of the 32 mg single intravenous (IV) dose and the restriction of the maximum single IV dose to 16 mg [2]. Concerning its safety during pregnancy, ondansetron remains controversial; therefore, additional research is required to fully comprehend the risks and benefits associated with its use [5]. We report a rare adverse effect of intravenous ondansetron in a primigravida that manifested as sinus bradycardia with progression to type 2 Mobitz second-degree atrioventricular (AV) block.

## Case presentation

A 26-year-old primigravida with gestational diabetes mellitus and hypothyroidism at 36 weeks and five days gestation was admitted for safe confinement. The patient had no prior cardiac illness. Because of the breech presentation of the fetus and delayed progression of labor, she was taken to the operating room for an emergency cesarean section. At admission, a normal sinus rhythm was noted on the ECG, with a PR interval of 200 milliseconds. Serum electrolytes were normal. The patient received pantoprazole 40 mg and ondansetron 4 mg intravenously. Following this, spinal anesthesia with 2 mL of 0.5% bupivacaine was administered. Just following induction, the patient’s heart rate decreased from 80 to 40 beats per minute; the rhythm was sinus, blood pressure was normal, and the patient was asymptomatic.

**Figure 1:**
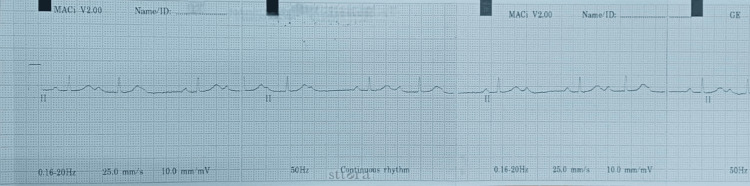
Electrocardiogram showing second-degree atrioventricular block: Mobitz type 1

Glycopyrrolate 0.2 mg intravenously was given, which raised the heart rate to 82 beats per minute. She underwent the planned cesarean section and delivered a female baby weighing 2.7 kg. As per the standard of care, she received an injection of ondansetron 4 mg to prevent postoperative nausea and vomiting. The cardiac monitor showed rhythm disturbances during the recovery period and an electrocardiogram with a rhythm strip revealed a second-degree AV block, Mobitz type 1 ( Figure 1), which persisted despite the patient’s diligent isometric exercises (which were tried to see if it could abolish the heart block).

The patient was shifted to the ICU for continuous cardiac monitoring. Interval ECGs taken over 12 hours showed a progression of the heart block, from type 1 Mobitz to type 2 Mobitz second-degree AV block.

The patient was administered ondansetron unsuspectingly for an episode of vomiting in the ICU. The patient underwent 24 hours of Holter monitoring which showed a Mobitz type 2 block. Echocardiography showed normal chamber dimensions with no regional wall motion abnormality. Ondansetron was suspected as being the cause of the heart block and further doses were not given. Holter monitoring performed for 48 hours after stoppage of ondansetron showed no evidence of heart block.

Outcome and follow-up 

The patient exhibited no symptoms during the remaining course of the hospital stay, and serial ECGs showed no heart block; hence, she was subsequently transferred to the ward and eventually discharged on day eight following hospitalization. On a review visit 15 days post discharge, the patient continued to be asymptomatic, and the repeat ECG done was normal. The patient continues to be asymptomatic to date. With no evidence of heart block on the admission ECG and normal thyroid and electrolyte levels, the transient heart block was attributed to the 5HT3 blocker ondansetron.

## Discussion

To prevent vomiting and nausea after surgery, ondansetron is often prescribed to block serotonin from interacting with the 5HT3 receptor [6]. Therapy with this drug is inexpensive and has a low risk of side effects such as headache, dizziness, and constipation [7]. The most worrisome side effect is QTc-prolongation, and clinicians should avoid these medications in patients with known prolonged QTc [8]. Although its clinical safety has been established by large-scale studies [1,9,10], there have been reports of myocardial infarction and arrhythmias such as supraventricular tachycardia, ventricular tachycardia, and atrial fibrillation [11]. A small number of cases of dysrhythmia have been reported following dosing with 4 mg of ondansetron [12]. Coronary vasoconstriction and inhibition of the Bezold-Jarisch cardiac reflex are two of the described mechanisms that may lead to rhythm disturbances after 5HT3 receptor antagonists [13].

Ondansetron's pharmacological effects on the heart are mediated by potassium channel-related mechanisms. Na+ channel opening is normally required for action potential upstroke (Phase 1) in the atria, Purkinje fibers, and ventricular cells. At this stage of the action potential (Phases 1 and 2), the Na+ current has been turned off, the calcium current (mostly of the "L" type) is fluctuating, and the K+ current is just starting to develop. In Phase 3, the Na+ and Ca++ channels are completely inactivated, and the permeability to K+ increases, resulting in a full repolarization. In this phase, fast- and slow-acting potassium ion channels play important roles, with the former playing a crucial role in the third. Prolonged cardiac repolarization has been attributed to ondansetron, which has a sub-micromolecular affinity for the K+ channel encoded by the human ether-a-go-go-related gene (HERG) [14,15].

Other 5HT3 blockers such as granisetron and dolasetron, can also cause ventricular arrhythmias by prolonging the QRS or QT interval through their effects on Na+ and K+ channels [1]. Nonetheless, the underlying cause of this bradycardia remains unclear. This is likely because of the multifaceted nature of the cardiovascular effects of serotonin receptors, which can include bradycardia, tachycardia, hypotension, hypertension, and vasoconstriction or dilatation [16]. Therefore, the effects of ondansetron, a drug that blocks 5HT3 receptors, will vary from patient to patient based on the level of preexisting serotonergic activity in the parasympathetic and sympathetic branches of the autonomic nervous system [12].

In a similar scenario described by Kadni et al., a 25-year-old woman gravida 2, Para 1, and a history of cesarean section was electively posted for the same, and she had bradycardia after receiving IV ondansetron 4 mg to prevent vomiting and nausea. During the ECG monitoring, ectopic atrial and ventricular activity was detected [17]. Similarly, ventricular tachycardia and ectopics were described as rare adverse effects of ondansetron in a case report involving a 24-year-old female undergoing a cesarean section [6]. Patients with electrolyte imbalance (hypokalemia, hypomagnesemia, long QT, bradyarrhythmia, and congestive cardiac failure) are advised against taking ondansetron, according to a warning issued by the US FDA [4]. When ondansetron was taken in high cumulative doses (24 mg, 32 mg), this effect became apparent. Cardiovascular disease patients, thyroid disease patients, the elderly, alcoholics, people with long-term hypertension, and those with a family history of atrial fibrillation are also at risk for drug-induced arrhythmias. Postoperative nausea and vomiting can also be treated with other medications, such as promethazine, scopolamine, diphenhydramine, and metoclopramide; however, they are not used because of adverse effects such as dry mouth, sedation, hypotension, extrapyramidal symptoms, dystonic effects, and restlessness [18,19].

## Conclusions

It is important to know that there is a rare but possible risk of encountering cardiac rhythm disturbances following intravenous administration of ondansetron. A new-onset rhythm disturbance after the administration of parenteral ondansetron should prompt the clinician to suspect an ondansetron-induced heart block along with other common causes of rhythm disturbances, such as hypokalemia, hypomagnesemia, cardiac ischemia, and hypoxia. The authors advocate for a cautious approach to using this medication. As soon as it is suspected that a drug may cause uncommon adverse effects, it should be stopped, and the patient's medical record should be updated to reflect this.
